# Surface effects of vapour-liquid-solid driven Bi surface droplets formed during molecular-beam-epitaxy of GaAsBi

**DOI:** 10.1038/srep28860

**Published:** 2016-07-05

**Authors:** J. A. Steele, R. A. Lewis, J. Horvat, M. J. B. Nancarrow, M. Henini, D. Fan, Y. I. Mazur, M. Schmidbauer, M. E. Ware, S.-Q. Yu, G. J. Salamo

**Affiliations:** 1 Institute for Superconducting and Electronic Materials, University of Wollongong, Wollongong, New South Wales 2522, Australia; 2 Electron Microscopy Centre, University of Wollongong, Wollongong, New South Wales 2522, Australia; 3 School of Physics and Astronomy, Nottingham Nanotechnology and Nanoscience Center, University of Nottingham, Nottingham NG7 2RD, United Kingdom; 4 Department of Electrical Engineering, University of Arkansas, Fayetteville, AR, 72701, USA; 5 Institute for Nanoscience and Engineering, University of Arkansas, Fayetteville, AR 72701, USA; 6 Leibniz-Institute for Crystal Growth, Max-Born-Str. 2, D-12489 Berlin, Germany

## Abstract

Herein we investigate a (001)-oriented GaAs_1−*x*_Bi_*x*_/GaAs structure possessing Bi surface droplets capable of catalysing the formation of nanostructures during Bi-rich growth, through the vapour-liquid-solid mechanism. Specifically, self-aligned “nanotracks” are found to exist trailing the Bi droplets on the sample surface. Through cross-sectional high-resolution transmission electron microscopy the nanotracks are revealed to in fact be elevated above surface by the formation of a subsurface planar nanowire, a structure initiated mid-way through the molecular-beam-epitaxy growth and embedded into the epilayer, via epitaxial overgrowth. Electron microscopy studies also yield the morphological, structural, and chemical properties of the nanostructures. Through a combination of Bi determination methods the compositional profile of the film is shown to be graded and inhomogeneous. Furthermore, the coherent and pure zincblende phase property of the film is detailed. Optical characterisation of features on the sample surface is carried out using polarised micro-Raman and micro-photoluminescence spectroscopies. The important light producing properties of the surface nanostructures are investigated through pump intensity-dependent micro-PL measurements, whereby relatively large local inhomogeneities are revealed to exist on the epitaxial surface for important optical parameters. We conclude that such surface effects must be considered when designing and fabricating optical devices based on GaAsBi alloys.

Recent advances in semiconductor growth technologies have seen III-V-Bi alloys—containing both semiconductor and semimetal components—emerge as a promising new family of semiconducting materials, attracting scientific and technological interest worldwide. For the case of dilute bismide compounds GaAs_1−*x*_Bi_*x*_^1^,^2^, bismuth induces an anomalously large and temperature-insensitive bandgap reduction, making them very appealing for optical devices accessing wavelengths in the infrared^1^,^3^,^4^,^5^. The interesting changes made to the host GaAs bandstructure mainly arise from the massive disparity in atomic size between group-V Bi and As atoms. Such a large difference also imposes fundamental challenges for Bi inclusion, whereby the growth of high-quality GaAsBi films with relatively large Bi concentrations is restricted to a relatively narrow growth window^6^. Namely, Bi is prone to segregate and form metallic droplets on the epitaxial surface during Bi-rich growth^7^,^8^,^9^,^10^,^11^. The surface-segregation of Bi during growth has motivated intense research into how to grow device-quality GaAs_1−*x*_Bi_*x*_ materials while retaining appreciable Bi incorporation^12^. Conversely, in the pursuit of realising record GaBi molar fractions (*x* = 0.22), the attitude of researchers toward the optical and structural quality of Bi-rich films appears more relaxed^13^.

Since Wagner and Ellis^14^ first reported on the vapour-liquid-solid (VLS) mechanism, it has been employed extensively to create a wide range of semiconductor nanostructures^15^. In the liquid phase, bismuth droplets are known to act as catalysts to the formation of GaAs-based nanostructures^16^,^17^,^18^,^19^, via the VLS mechanism. Knowledge of the destabilisation process^16^ and the movement of the Bi droplets (sometimes referred to as crawling, self-propagating or running), as well as the subsequent formation of in-plane structures, is vital to form a complete understanding of GaAsBi compound growth.

Investigations utilising elemental bismuth to seed the VLS growth of III-V nano- or microstructures are limited^16^,^17^,^19^, with studies often tending to focus on alternative material systems; Cd(Se,Te)^20^, PbTe^21^, SnS_2_^22^ and Si^23^ nanowires. Bismuth is a group V species, however it does not readily form a solid binary compound with Ga. Rather than being directly harmful to the electronic properties of the host, like silver- or gold-seed atom incorporation^24^,^25^, bismuth alters the GaAs bandstructure^26^,^27^,^28^. Thus the small amount of scientific interest in Bi-assisted III-V nanostructure growth using the VLS mechanism is surprising, given its candidature as an alternative “self-seeded” type of catalyst, able to tune nanowire properties^29^ through intentional incorporation of Bi atoms from the catalyst. The bismuth-initiated growth of III-V (InAs, GaP, GaAs, and InP) nanowires has already been realised by employing the solution-liquid-solid (SLS) technique^30^. Since a solution-based synthesis differs considerably from a vapour-based method, it is difficult to directly compare these findings with the current work. However, it is worth mentioning that the nanowires grown by SLS demonstrated good crystal quality, with few stacking defects^30^.

Besides a large number of investigations into GaAsBi quantum well structures and some more recent work on quantum dot-like structures^31^,^32^,^33^, detailed studies of GaAsBi nanostrucutres are limited^17^,^19^,^34^,^35^,^36^. Sterzer *et al*.^34^ showed that for static Bi surface droplets the crystalline Bi exhibits preferential lateral ordering with respect to the GaAsBi surface after cool-down. A more recent study by Essouda *et al*.^19^ reported the first synthesis of Bi-catalysed GaAsBi nanostrucutres atop GaAs, grown by atmospheric pressure metalorganic vapour phase epitaxy. Here, simple fully-tapered planar structures were synthesised, however none reached over a micron in length. Further, they did not investigate the structural details of their nanostructures, nor their important optical properties. Ishikawa *et al*.^36^ recently reported the synthesis of vertical GaAs/GaAsBi coaxial multishell nanowires on a Si substrate, initiated by constituent Ga-induced VLS growth, rather than Bi-induced, for realizing III-V nanowires able to access the near-infrared spectral range. Very recently, we reported on the Bi-seeded growth of GaAsBi planar nanostructures exhibiting interesting periodic height variations^17^. While the present paper was under revision, a study was published by Wood *et al*.^37^ that reported on in-plane GaAs nanowires embedded into a (001) GaAsBi film grown by molecular-beam-epitaxy (MBE). The work focused on cross-sectional transmission electron microscopy (TEM) experiments to describe the morphology and compositional details of their planar nanostructures and proposed a growth model that was mediated by their Ga-rich MBE reactor conditions. Currently, excluding theoretical work into GaAsBi nanowires^35^ and quantum dots^32^, these few recent studies^17^,^19^,^34^,^36^,^37^ encompass the full degree of literary work on the subject.

Several investigations have done well to identify the MBE growth conditions that lead to droplet formation^7^,^8^,^9^,^10^,^11^, however surprisingly little is known about the mechanisms governing their kinetic behaviour or their potentially disruptive interaction with the underlying crystal. In most cases, experimental probes are far larger than the dimensions of the droplets/tracks on the surface (order of nm to *μ*m^7^,^8^,^9^,^10^,^11^,^17^,^19^,^34^), with measurements unavoidably recording an unresolved average of the entire probed area. While the removal of Bi surface droplets post growth (through selective wet chemical etching), and prior to characterisation, appears to rid the Bi metal, a number of experimental studies^38^,^39^,^40^ have neglected to consider their lasting impact on the structural and optical properties of the epitaxial surface. This issue we investigate in the present study using electron microscopy techniques and micro-optical probes (lateral resolution ~1*μ*m).

## Experimental Procedure

### Sample details

**Figure 1:**
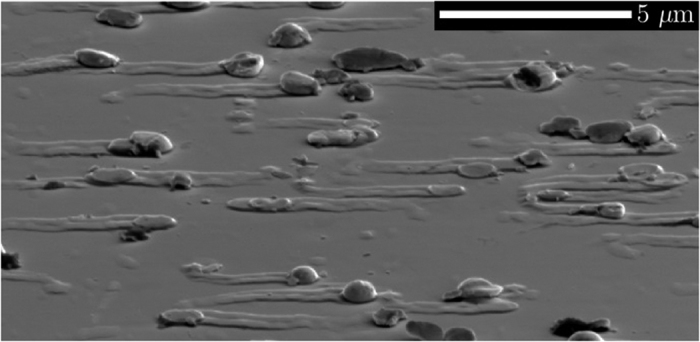
Droplet covered GaAsBi surface. SEM image of GaAsBi sample surface, exhibiting surface droplets and the formation of self-aligned trailing nanotracks.

The (001) GaAsBi/GaAs sample investigated was grown by MBE with a relatively large Bi flux which was well within the Bi saturation regime^8^, whereby an alloying limit is imposed by the low miscibility of Bi into GaAs. As a consequence, liquid Bi droplets formed (see Fig. 1) through aggregation of unincorporated Bi atoms on the surface. *Ex situ* EDS experiments revealed that the surface droplets were indeed pure Bi, with no observations of dual phase-separated Ga-Bi droplets^37^, or any pure Ga deposits present on the surface. As an essential requirement for the MBE of GaAsBi, growth is carried out at the relatively low substrate temperature (compared to the MBE of other III-V compounds) of 325–330 °C. This is well above the Ga-Bi eutectic point (222 °C) and low melting point (compared to other group-V metals) of pure Bi metal (271 °C). During growth, planar nanostructures (including nanotracks reaching approximately 50 nm in height and 4 *μ*m in length) were synthesised on the GaAsBi epitaxial surface in parallel to the MBE of a ~220-nm thick GaAsBi epitaxial layer. The formation of the droplets and trailing nanotracks on the GaAsBi surface is exhibited in Fig. 1. The final GaAs_1−*x*_Bi_*x*_ epilayer was found to be compositionally graded into two halves of approximately equivalent thicknesses. The nominal bismuth concentration in the top GaAs_1−*x*_Bi_*x*_ epilayer is *x* = 4.1%, while the subsurface layer contained a Bi concentration of *x* = 2.5%, determined through a combination of evaluating both high-resolution x-ray diffraction (HRXRD) data^2^ and the micro-photoluminescence (micro-PL) peak energy^41^. The Bi concentration measured in this way is found to be consistent across the droplet-free regions of the epitaxial surface, and comparable to known Bi saturation limits for GaAs_1−*x*_Bi_*x*_ alloys grown under similar conditions^42^. Further growth details can be found in ref. 17.

For the TEM experiments reported in this study, a cross-sectional lift out of the sample surface was performed using a FEI Helios 600 NanoLab DualBeam focused ion beam (FIB)/SEM system. A prominent planar nanostructure was selected for lift out with the sample prepared via the FIB method constituting the longitudinal axis of a 4.5 *μ*m subsurface nanowire. Prior to employing the FIB (Ga ions) an initial layer of a mixed Pt and C composite was deposited onto the surface, followed by a relatively thick deposition of pure Pt, to prevent modification occurring (structural changes, Ga corruption, etc.) on the surface due to the milling and sample preparation. The final thickness of the specimen was reduced through milling to less than 80 nm.

### Characterisation details

Both room-temperature micro-Raman scattering and micro-PL spectra were acquired in a quasi-backscattering configuration on the (001) sample surface using a confocal Jobin-Yvon HR800 integrated system, employing a 20 mW HeNe 632.8 nm laser for excitation. Raman and PL signals were recorded using an air-cooled CCD and a liquid nitrogen-cooled InGaAs detector, respectively. The implementation of an Olympus x100 objective defined an approximately 1*μ*m diameter Gaussian optical microprobe and laser power densities were controlled by a neutral variable density filter situated before the microscope optics.

High-resolution x-ray diffraction (HRXRD) was carried out with a commercial x-ray diffractometer (GE Inspection Technology). A combination of a precollimating parabolic multilayer mirror and a two-bounce Ge 220 channel-cut monochromator was used to select the Cu K*α*_1_ line at *λ* = 1.54056 Å and to collimate the incident x-ray beam to about 0.007°. Primary slits were employed to define the size of the incident x-ray beam at the sample to 0.3 mm × 5 mm. X-ray rocking curves (2:1 scans) were recorded with a single channel scintillation detector which is equipped with 0.3 mm × 5 mm receiving slits. Reciprocal space maps (RSMs) were recorded using a linear position sensitive detector, which simultaneously measured the direction of the diffracted beam with an accuracy of about 0.01°. The RSMs were collected by a single rocking scan of the sample while the detector was kept fixed ensuring fast data collection combined with very good counting statistics. For further details of this technique, see ref. 43.

The scanning electron microscopy (SEM) and energy-dispersive x-ray spectroscopy (EDS) data was acquired using a JEOL JSM-7001F instrument in conjunction with an Oxford Instruments X-max 80 energy dispersive x-ray spectrometer. The high stability Schottky field emission source permits the acquisition of both high resolution x-ray data and electron images (~1.2 nm). Elemental analysis through EDS does not lend itself to the absolute determination of semiconductor alloy compositions with very low concentrations of alloying material, as it usually involves significant components of scaling and relative error in addition to unpredictable surface effects. Thus, semi-quantitative EDS analysis was used to confirm the local composition of the nanostructure relative to the epitaxial film (absolute composition determined independently through HRXRD and PL analysis). A 200 kV probe corrected JEOL JEM-ARM200F scanning transmission electron microscope (STEM) was employed for high-resolution structural characterisation. This instrument is capable of atomic scale imaging and features a resolution of <0.08 nm. STEM micrographs were recorded in both annular bright-field (BF) and dark field (DF) detection modes. Recorded images were post-processed for fast Fourier transform (FFT) and geometrical phase analysis (GPA) by respectively using the Digital Micrograph software (GATAN Inc., Pleasanton, CA, USA) and Crystallographic Tool Box (CrysTBox) add-on for MATLAB^44^.

## Results and Discussion

### Structural and compositional characterisation of the GaAsBi epitaxial film

**Figure 2:**
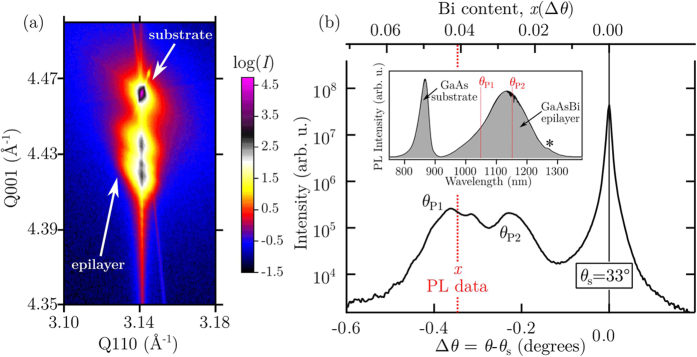
HRXRD characterisation of (001)-oriented GaAsBi/GaAs heterostructure. (**a**) X-ray reciprocal space map in the vicinity of the asymmetrical 224 reciprocal lattice point of the GaAs substrate indicating coherent growth of the GaAsBi epilayer. The diffuse scattering nearby the epilayer reflection is caused by structural defects in the film (**b**) High-resolution x-ray rocking curve (2:1 scan) around the 004 symmetrical Bragg reflection of the GaAs substrate showing a broad feature from the GaAsBi epilayer. The broken vertical line in the x-ray data relates to the strained Bi composition axis (top axis) and indicates the expected Bragg angle for a epitaxial Bi composition of 4.1%, extracted^41^ from the PL data shown in the inset. Likewise, the two broken vertical lines in the inset are derived in a similar fashion, determined from the HRXRD data by applying Vegard’s law and using *x*_Strained_ = 6.77 × Δ*θ*. Here ‘*’ indicates a spectral artefact in the PL data.

In order to investigate the strain state of the epitaxial film an x-ray reciprocal space map (RSM) has been recorded around the asymmetrical 224 GaAs reciprocal lattice point, as shown in Fig. 2(a). The RSM contains scattering from both the GaAs substrate (top peak) and multiple peaks from the GaAsBi epitaxial film experiencing vertical lattice expansion (lower peaks). The horizontal component of the scattering vector, Q_110_, is identical for both substrate and film reflections, indicating completely coherent growth of the GaAsBi layer on the GaAs substrate, with no substantial plastic relaxation. However, the existence of weak diffuse scattering in the vicinity of the epilayer peaks indicates the presence of structural defects.

**Figure 3:**
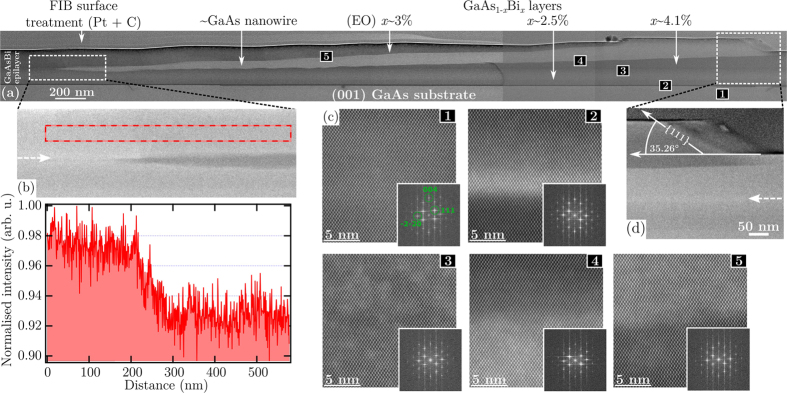
TEM characterisation of epitaxially buried nanowire structure. (**a**) Stitched BF TEM survey micrograph of a representative buried nanostructure imaged across the longitudinal cross-section, revealing the presence of a complex subsurface nanostructure initiated mid-way through growth at a compositional step. The various compositions displayed are determined through a combination of EDS, HRXRD and PL analysis. (**b**) Normalised intensity line scan (bottom) of a HAADF (Z-contrast) TEM image (top) recorded at the start of the nanowire, as indicated in (**a**). (**c**) HAADF TEM images recorded along the [110] zone axis at the five locations indicated in (**a**), with the insets showing the results of the FFT image reconstructions. (**d**) HAADF TEM image recorded at the termination of the nanowire growth, as shown in (**a**), with the angle indicating the front growth facet belonging to the {111} family. The dashed arrows in the Z-contrast images contained in (**b**,**d**) highlight the Bi composition step position, appearing approximately half way through the epitaxial layer.

Figure 2(b) presents the x-ray rocking curve (2:1 scan) around the 004 symmetrical Bragg reflection. Along with the sharp 004 GaAs substrate reflection appearing at *θ*_s_ = 33.026°, a broad feature is observed at negative Δ*θ* values – relative to the GaAs substrate – indicating scattering from a vertically expanded lattice, caused by the Bi alloying. Wide angle XRD scans (data not shown) revealed weaker peaks at approximately 27°, 38°, and 46°, confirming the presence of crystalline Bi on the sample surface. For a pseudomorphically grown epilayer such as ours, multiple peaks at negative Δ*θ* suggest the presence of compositional steps (grading) within the film. In fact, from cross-sectional TEM and EDS measurements (see Fig. 3(a,d)), such a layered structure is revealed to exist in our sample. Out-of-plane lattice distortions due to the Bi alloying can be determined experimentally from the HRXRD scan through^45^


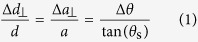


The ratios Δ*d*_⊥_/*d* and Δ*a*_⊥_/*a* are defined as the relative distance changes between the lattice planes and lattice constants, respectively, while the measured angular shift is given by Δ*θ* = *θ* − *θ*_s_. The relaxed lattice parameter in the epitaxial film is calculated by


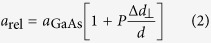


We assume the deformation constants of our dilute GaAsBi alloy are the same as those for pure GaAs and, apply Vegard’s law, obtain an estimation for the Bi content from the HRXRD data using


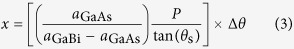


Here *a*_GaBi_ and *a*_GaAs_ indicate respectively the lattice constants for the GaBi (6.33 Å^2^,^38^) and GaAs (5.65 Å), while *P* represents the distortion coefficient. For the case of a psuedomorphic dilute GaAsBi film, we input the pure GaAs distortion coefficient via the elastic constants; *P* = *C*_11_/(*C*_11_ + 2*C*_12_) = 0.527. It follows that for a fully strained GaAs_1−*x*_Bi_*x*_ epilayer, the Bi concentration can be estimated by the linear relationship *x*_Strained_ = 6.77 × Δ*θ*. This relation forms the basis of the top axes in Fig. 2(b).

In Fig. 2(a,b) multiple diffraction peaks are observed for the GaAsBi epilayer due to the coherent superposition of partial waves from the graded areas inside the layer. Similar features can be found in smoothly graded coherent films, in that even a smooth grading could lead to the appearance of multiple peaks. In particular, their appearance does not necessarily point to discrete steps of Bi composition. Moreover, coherent Pendellösung fringes caused by the finite thickness of the epilayer may show up which further handicaps a straightforward interpretation of the x-ray data. An accurate and meaningful HRXRD simulation is hindered by the complex nature of our structure: epitaxial growth of a three dimensionally structured film (see, for example, Fig. 3(a)). Nevertheless, the angular positions of the diffraction peaks can be used to estimate the corresponding out-of-plane lattice parameters and provide Bi compositions through Eq. 3.

Examining the data shown in Fig. 2(b) three main peaks are identified; a sharp and high intensity peak located at 0° (*θ*_s_) coming from the (001) GaAs substrate, and two broader low intensity peaks (*θ*_p1_ and *θ*_p2_) located at negative Δ*θ*, originating from the strained GaAsBi epitaxial lattice. Besides the two peaks relative to the layer and substrate diffractions, the scan shows Pendellösung oscillations (or “thickness fringes”), which are seen to strongly occupy the wings of *θ*_p1_, due to the presence of a smooth and coherent growth interface. Further, in corroboration with TEM measurements, the width and intensity of the weaker peak positioned between *θ*_p1_ and *θ*_p2_ strongly suggests it originates from such an intensity oscillation; both the TEM data and the width of the Pendellösung oscillation indicate an epitaxial thickness of ~200 nm. Observations of intermediate peak splitting are not uncommon to GaAsBi/GaAs epitaxial structures^11^ and are typically present in epitaxial layers with an inhomogeneous composition step, or in the presence of lattice relaxation. Assuming a fully strained alloyed system and the peaks are due to different Bi contents in the GaAsBi layer, the peak angular separation measurements give two values; *θ*_p1_ is relative to a Bi composition of 4.2%, while *θ*_p2_ is nearly half that at 2.5%.

Due to the shared ability of PL^41^ and XRD experiments^2^ to evaluate the Bi molar fraction in GaAs_1−*x*_Bi_*x*_, a comparison of the two techniques is displayed via the inset in Fig. 2(b) and the dashed (red) vertical lines, which indicate their complementary findings. Interestingly, a single unambiguous peak is observed in the PL data recorded from the sample surface away from the nanotracks, providing a Bi composition estimate of *x* ~ 4.1%. Micro-PL measurements performed at an angle of 60 degrees to the surface normal showed no modification to this peak while expectedly suppressing the relative substrate signal, and confirmed the emission to arise from the surface of the GaAsBi epitaxial film. The absence of a clear third PL peak—expected to manifest at 1049 nm from a Bi composition of *x *= 2.5% —leads one to believe that its signal is beyond the detection limits of our instrument. There are a number of influences which may govern this. Oe^1^ has associated no PL response (non-radiative recombination) to a compositional phase separation in the ternary system. Moreover, together with the fact that PL intensities have been shown to strongly correlate with the Bi molar fraction (up to ~4%^41^) and that the bandgap energy of the top most layer will be smaller than the bottom layer, it is not surprising a PL emission arising from the bottom compositional step within the epilayer is not observed. Thus, hereon we do not consider PL emissions from the subsurface layer containing a Bi contnet of 2.5%.

The discrepancy between the two methods for determining the Bi content in the top layer of the film is yet to be discussed; the position of *θ*_p1_ possesses an additional angular shift of Δ*θ* *=* −0.018°, compared to that expected from analysing the PL peak energy. Observations of negative peak splitting in GaAs epilayers grown by MBE at low temperatures are well documented, and characterized by excess As in the form of antisites (As_Ga_). Large populations of As_Ga_ introduce compressive strain, and is detectable through XRD measurements^46^. The formation of As_Ga_ during the MBE of GaAsBi were shown to proliferate for growth temperatures below 315 °C^47^, while strongly suppressed above. That our sample is grown at a substrate temperature of 330 °C, negates such a defective presence. More likely is the existence of significant Bi clustering within the GaAsBi matrix^46^ which give rise to relatively high free p-type carrier populations^48^, introduced by Bi-induced acceptor states^49^. This is evidenced in our nominally undoped sample through observations of a strong damped LO-phonon-hole-plasmon coupling (LOPC) mode in the Raman spectra measured from the film surface, with analysis^50^ concluding a relatively large native hole concentration in the order of *p* ~ 10^18^ cm^−3^. Given our epilayer is relatively thick (~200 nm), we suggest that inherent structural disorder and/or Bi clustering could contribute to the additional angular shift observed in the HRXRD data. At present, a detailed investigation on the effects which Bi clustering has on XRD measurements of lattice spacing is lacking, preventing comparison. For this reason, while the Bi contents determined through HRXRD (*x* = 4.2%) and PL (*x* = 4.1%) exhibit great agreements, we assign the PL-derived estimate of *x* = 4.1% for the top layer.

Figure 3(a) presents a cross-sectional BF TEM image of our GaAsBi/GaAs epitaxial structure, focusing on the morphology and composition of a representative planar nanostructure imaged across its longitudinal axis. Examining the regions free from the formation of nanowire material on the far left and right of the stitched micrographs, the contrasting difference between the top and bottom of the GaAsBi epitaxial film confirms a graded two layer structure, in support of the model employed to interpret the HRXRD data. From the perspective offered in Fig. 3(a), the entire in-plane nanowire is revealed to be subsurface, initiating growth—in the region enlarged in Fig. 3(b)—roughly midway through the MBE of the film and rising up toward the surface throughout.

The Bi content values displayed in Fig. 3(a) have been calculated by combining EDS measurements with the aforementioned results of the HRXRD and PL analysis. The measured EDS signal, as well as the Z-sensitive high angle annular dark field (HAADF) TEM signal, exhibited a tendency to steadily decay when recorded near the top of the MBE growth surface. Such a change coinciding in both signals is likely due to a systematic variation in the sample thickness in this direction, as it tapers off toward the top of the image, caused by the FIB lift out processing. Conveniently, to account for this in our semi-quantitative EDS analysis, we rely on the fact gallium atoms should always hold a 50% relative compositional weight throughout a GaAs_1−*x*_Bi_*x*_/GaAs heterostructure. It follows that the ratio of the Bi-related EDS signal to that detected from Ga atoms will provide a reliable assessment of local composition as they will both depend on the sample thickness. Finally, the ratio of raw Bi and Ga EDS signals were rescaled to align with known Bi content values determined using HRXRD and PL analysis. It should be pointed out that the independent finding of all techniques displayed excellent agreement. Micro-PL characterisation of the nanotrack is presented later.

Evaluating the EDS-obtained Bi content of the regions probed in Fig. 3(a) multiple well-defined deposits of GaAs_1−*x*_Bi_*x*_ alloy were found to exist in the graded epilayer, nanowire and surrounding material; Bi molar fractions are displayed along the top of Fig. 3(a). Two of these regions relate to the bottom (*x* = 2.5%) and top (*x* = 4.1%) halves of the compositionally graded epitaxial film growth. A third alloy composition is defined here by epitaxial overgrowth (EO) and resides above the approximately pure GaAs nanowire, persisting its full length. While the incorporation of some Bi atoms into the nanowire is inevitable, we find that within the experimental error the Bi alloying in the structure is trivial (likely in the realm of doping, rather than alloying). The Bi contents identified in Fig. 3(a) are found to be consistent across the whole structure, as well as the Bi profile measured on another FIB prepared sample (data not shown). This suggests the Bi incorporation efficiency for GaAsBi alloy growth above the nanowire is consistently reduced relative to the surrounding smooth film by 25%, generating a significant compositional inhomogeneity across the surface for all nanotracks.

While bright field (BF) TEM is often implemented for large magnification imaging and offers high resolution, it does not offer the contrast required to classify our sample effectively. Because it is highly sensitive to variations in the atomic number of atoms, HAADF TEM is better suited to probe—at the nm-scale—the compositional difference between regions containing differing amounts of high-Z Bi^37^ within the GaAsBi/GaAs heterostructure. To highlight the significantly divergent nature of the Bi alloying above the nanowire (forming the EO), the top image in Fig. 3(b) presents an enlarged HAADF TEM micrograph recorded across the start of the nanowire, along with a corresponding intensity line scan plotted on the bottom. The distinct drop in the intensity line scan here indicates a reduction in the Bi alloying across the area scanned. The growth and environment conditions for the synthesis of GaAsBi atop the nanowire, forming the EO layer, will inherently differ from the surrounding undisturbed epitaxy process. Specifically, a difference in growth rate is reflected in the rise of the above-surface nanotrack compared to the rise in the buried nanowire, which is not 1:1; the growth EO crystal proceeds relatively slowly. Interestingly, at approximately half way through the MBE of the GaAsBi film, there appears a thin Bi-rich region which coincides with the beginning of the Bi droplet-mediated nanowire growth. For reference, this region is highlighted by the dashed arrows contained in Fig. 3(b,d). Under constant MBE reactor conditions throughout growth, the cause of the formation of a Bi-rich region is unclear. Ultimately its consequence was to initiate the growth of the planar nanowires via Bi surface segregation, and the formation of Bi droplets capable of catalysing the growth of planar nanowires. In parallel to the growth of a rising epitaxial film, the Bi droplets deposited a buried and diagonally rising nanowire.

Investigations into the microstructure of the sample cross-section are carried out by the employing HAADF high-resolution TEM (HRTEM) modality, which directly reflects changes in the crystal structure. Figure 3(c) presents representative HAADF HRTEM images of the growth interface (for example, the GaAsBi(epilayer)/GaAs(nanowire) boundary/interface investigated at^4^) contained in Fig. 3(a) and, for reference, the GaAs substrate^1^. These images permit the valuable assessment of crystal defects and potential phase aberration across the compositional growth boundaries within the structure. In agreement with our HRXRD analysis, the atomic alignment portrayed here indicates the entire structure is coherently grown.

Due to the notoriously difficult task of achieving crystallographic phase purity in III-V nanowires, we examine next the phase details of our nanostructure and explore any lasting polytypes within the epilayer, caused by the presence of the embedded nanowire. III-V epitaxial nanostructures typically form in a cubic zincblende (ZB) crystal structure, though can preferentially adopt the hexagonal wurtzite (WZ) phase^51^,^52^ in cases of relatively small dimensional nanowires (tens of nm diameter)^53^,^54^. Size is not the only factor that allows such a structural transition, because the crystalline polytype may also be tuned by the use of appropriate growth conditions. Variability in the local phase expressed by the crystal is in fact strongly governed by several independent factors. The dynamical model described recently by Jacobsson *et al*.^55^ appears to be one of the most comprehensive available, accounting for interface morphology, chemical composition (bond ionicity), step flow and catalyst geometry, volume and wetting angle (at the triple phase boundary). With regards to our relatively large—dimensions in the order of hundreds of nm – in-plane GaAs-based nanostructures, the nanowires will epitaxially align their axes away from the surface normal direction, and the liquid Bi seed will remain in contact with both the (001) GaAsBi/GaAs surface and the faceted growth front (growth plane highlighted in Fig. 3(d)). The effects of this growth geometry will dominate the phase dynamics by significantly increasing the relative energy barrier for the formation of any crystal phase other than that of the epitaxial surface. It will also limit the size of the exposed surface available to accommodate the ZB to WZ alteration. The surface energy influences that promote the formation of a WZ phase in thinner nanowires are correspondingly reduced (or relatively absent).

Evidence within Fig. 3(c) of a marked ZB → WZ phase shift should take the form of facets becoming vertical, rather than diagonal. Vertical facets are not observed in these data. Moreover, the FFT pattern derived from these images should exhibit change in the presence of a WZ phase; this images we present in the respective insets of Fig. 3(c). Relative to the FFT image reconstruction generated from the GaAs substrate at^1^ in Fig. 3(a), no modifications attributable to the WZ polymorph are seen to arise at boundary locations^2^,^3^,^4^,^5^. This suggests the ZB phase is preferentially sustained throughout the entire coherently grown structure. This notion is further supported by the fact that a WZ phase is not detected by HRXRD or by micro-Raman backscattering characterisation (the WZ-related vibrational signature located at 255 cm^−1^ 56,57 was never recorded from the sample surface). Consequently, we determine a pure ZB phase to persist throughout our (001) GaAs_1−*x*_Bi_*x*_/GaAs epitaxial structure. While it is possible a WZ phase will form early on during nanowire growth, temporal changes to growth dynamics, namely, epitaxial burying^54^,^58^ and/or nanowire merging^53^, will see any embedded WZ crystal rapidly transition into the bulk ZB phase.

### The VLS mechanism and preferential growth

**Figure 4:**
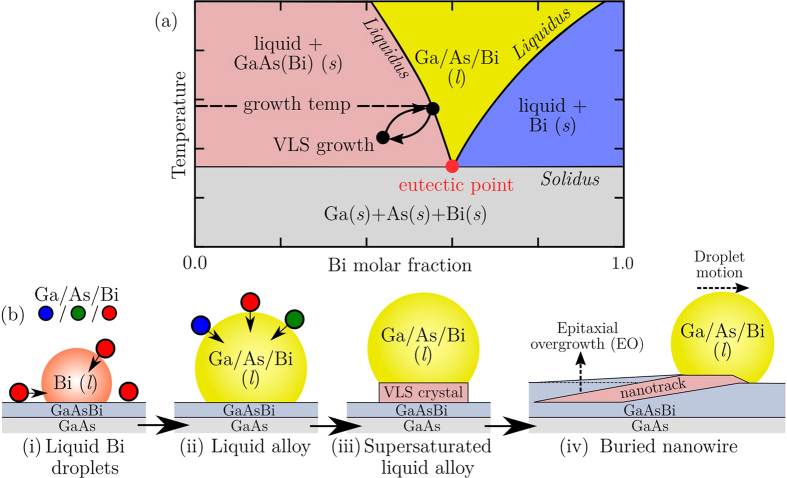
GaAsBi crystal growth via the VLS mechanism. (**a**) Illustration of the pseudobinary (Ga/As)-(Bi) eutectic phase diagram where, for clarity, the scheme has been simplified to treat both Ga and As components together, as one part of a binary system. Note that this plot does not portray actual data points (the true eutectic point is unknown, for example) and is presented purely for demonstrative purposes. Features of this diagram are described in detail in the text. (**b**) Schematic illustration of (i) Bi segregation and development of liquid surface droplets during MBE, (ii) the absorption of Ga and As species and the formation of a eutectic liquid alloy. (iii) The deposition of VLS crystal at the liquid-solid interface and (iv) the planar migration of the VLS-driven droplet during epitaxial overgrowth, resulting in a buried planar nanowire.

The use of liquid metals to mediate the growth of high-aspect ratio semiconductor nanostructures in a vapour environment is well established^14^,^15^,^59^,^60^. The general mechanism for VLS growth involving Ga, As and Bi is most easily described by considering the illustration of a simplified phase diagram (pseudobinary (Ga/As)-(Bi)) for the Ga-As-Bi eutectic system presented in Fig. 4(a). By its very composition and nature, a mixed eutectic system will exhibit a melting temperature far lower than that possessed by its pure (or diminutive alloy) constituents; the minimum melting temperature occurring at some well defined alloying ratio is defined as the *eutectic point*.

The liquidus line in Fig. 4(a) separates the all-liquid phase from the liquid + solid phases, while the horizontal solidus line separates the liquid + solid phases from the all solid (crystal) phase. Such phase boundaries are determined through experiment, whereby a systematic mapping of the alloy transition points is measured. We point out that such data is not presently available in the literature for the current *ternary* system. For changes in alloy composition, growth at a constant temperature defines where on the liquidus boundary the alloy phase will transition. VLS growth is indicated Fig. 4(a) by the cyclic arrows, in which the eutectic system transitions at the liquid-solid interface between an all-liquid state (gold region) and a state of liquid + GaAs(Bi) (*s*) (red region), by continual equilibration via VLS growth. Figure 4(b) presents a schematic of the different stages of the overall process leading to VLS growth of an embedded nanowire. Once surface-segregation produces Bi surface droplets, and in the liquid phase, the eutectic will absorb Ga and As species from a vapour with a higher chemical potential (*μ*_v_), relative to the liquid chemical potential (*μ*_1_). Thus the difference in these quantities is Δ*μ*_vl_ = *μ*_v_ − *μ*_l_, where the eutectic is supersaturated (concentration of the components in the liquid phase is higher than the equilibrium concentration; red region of Fig. 4(a)) for Δ*μ*_vl_ > 0. While supersaturated, the Bi eutectic droplet may lower its energy through VLS growth at the liquid-solid interface. The formation of crystal at the interface displaces the surface droplet and causes lateral motion which, in parallel to the formation of the epilayer, results in the embedment of the droplet catalysed nanowire in the film. Interestingly, many of the nanotracks examined in Fig. 1 terminate without the Bi surface droplet catalyst, a trait observed across the whole surface. Within the MBE reactor the pressure of Bi is high at 330 °C and a sufficiently large Bi beam equivalent pressure (BEP) is required to avoid total evaporation of Bi droplets. If the Bi BEP drops well below the Bi desorption rate, none of the bulk Bi will remain. That many nanowires terminate forming nanodiscs^16^ without droplets reflects a partial disruption of this fine balance towards the final stages of growth. Consequently, the terminating nanodisc, along with the whole nanowire, is then exposed to be buried by the growth of the rising epilayer and EO.

**Figure 5:**
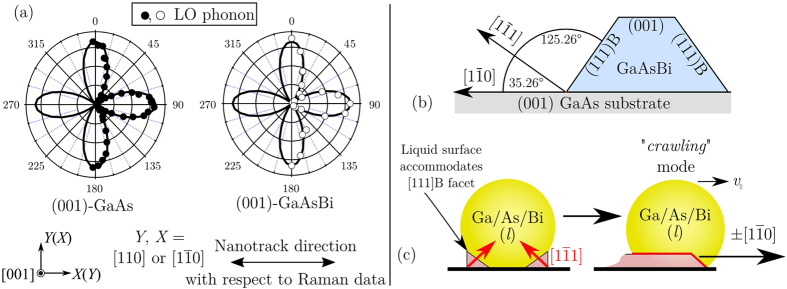
Determining the direction of preferential nanotrack growth. (**a**) Polarised micro-Raman backscattering LO phonon polar plots measured from pure a (001) GaAs crystal surface and the (001) GaAsBi surface. Below the plots are the corresponding possible basis directions (either [110] or 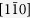) deduced from the analysis of the (001) GaAs polar data and the preferential nanotracks growth direction, measured with respect to (WRT) the GaAsBi polar plot. (**b**) Geometric information of relevant crystal faces relative to the [001] growth direction, and (**c**) a schematic illustration of two simultaneous [111]B growth planes accommodated by the liquid interface, which evolves ultimately into movement in a single planar direction. A “crawling” mode then propagates with in-plane velocity *v*_||_ in the ±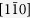 bi-direction, with the liquid droplet pinned on nanotrack top by surface tension.

Assessing the relative growth directions of the nanotracks contained in Fig. 1, there appears to be a common bi-directional axis in which they have formed. The formation of VLS crystal at the liquid-solid interface will typically favour facets with the lowest energies and the subsequent droplet motion will often be perpendicular to these crystal faces. The propensity for low-energy faces to grow more rapidly is the origin of the nanotrack self-alignment. We employ polarised Raman measurements to assist in determining the preferential direction of self-alignment. Figure 5(a) displays LO phonon polar plots of polarised micro-Raman backscattering recorded from a (001) GaAs and (001) GaAsBi surface, in a parallel configuration; 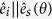, where *θ* defines sample rotation around the *c*-axis and parallel vectors 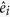 and 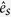 respectively indicate the polarisation of incident and scattered photons. Here the theoretical phonon intensities are also included, represented by the solid line in Fig. 5(a). Comparing the intensity of the LO band measured from the GaAsBi surface and from a pure (001) GaAs single crystal, one can conclude that the surface nanotracks align with either the ±[110] or ±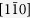 crystallographic directions.

For a zincblende GaAs-based system such as GaAsBi, the (111)B face has the lowest free energy^61^ and planar GaAs-based nanowires grown on GaAs (001) have been shown to share the same (111)B growth interface with vertical nanowires except the planar nanowire propagation direction is not orthogonal with the growth plane^62^. Relative to the (001) epitaxial growth surface, the growth facet plane shown in Fig. 3(d) is measured at ~35.3° and resides in the {111} plane family. Relative to the (001) epitaxial growth surface, the [111]B direction (also defined as [1
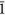
1]) is shown in Fig. 5(b) and agrees with the facet angle measured by TEM in Fig. 3(d). A dominant growth facet pointing in the ±[111]B direction posses a lateral projection of ±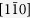 for in-plane motion. Thus, ±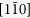 is the bi-direction we assign to our nanotracks. This directional assignment agrees well with other reports of self-aligned planar growths^63^,^64^,^65^,^66^. Figure 5(c) shows how lateral motion in the ±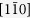 directions is achieved by this process; the formation of a flexible liquid surface at the liquid-solid interface facilitates preferential growth away from the *c*-axis, with the droplet motion restricted in-plane. With multiple (111)B facets simultaneously competing, the final direction of the crawling mode is eventually dependent on local topological or thermal fluctuations. Nanotrack growth then proceeds with the liquid Bi droplet held at its front by surface tension. It is interesting that we resolve self-alignment in these planar nanostructures. For reference, the Ga-mediated trails reported by Wood *et al*.^37^ adopted a less uncommon [110] growth axis, along with a lens-like V-shape through their transverse cross-section. This cross-sectional shape has been shown to associate with the [110] growth axis^62^, as the geometry permits the nanowire to retain a boundary with the [111]B side facets throughout growth. Investigations of the transverse cross-section of our buried nanowires (data not shown) does not reveal this features, rather they exhibit a simple flat bottom surface.

### Micro-optical characterisation of GaAsBi surface features

As outlined in the introduction, it is important to determine local deviations in the epitaxial properties arising in the presence of VLS-driven Bi surface droplets. Without the intricate preparation of a sample cross-section (like the preparation of the sample cross-section investigated by TEM previously), top-down methods such as HRXRD and electron diffraction techniques possess the wrong interaction volumes to reliably probe *localised* surface structure. In this section, we examine the microstructure and important optical properties of several Bi-mediated nanostructures present on the sample surface by employing room-temperature micro-Raman and micro-PL measurements.

#### Optical penetration of micro-Raman and micro-PL signals

We assess here the optical penetration depth, *d*_opt_, of the micro-Raman and micro-PL measurements performed using our integrated system on the sample surface. This is because if the optical penetration depth exceeds the thickness of the structures studied, a non-trivial subsurface signal may contribute to the recorded Raman or PL spectrum. Our Raman and PL measurements both employ 632.8 nm HeNe laser light for excitation, however the two techniques have very different interaction volumes. Typical depth analysis is determined by considering the absorption coefficients for both the incoming (*α*_in_) and the outgoing (*α*_out_) light:


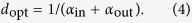


Raman backscattered photons (with only a slight energy shift) from a material with absorption coefficient *α* ≠ 0 will only possess a *d*_opt_ half of the light penetration; *d*_opt_ = 1/2*α* (*α* = *α*_in_ ≈ *α*_out_). For pure GaAs, Raman is able to provide information from a depth of slightly larger than 100 nm, with the majority of the exponentially decaying signal arising from the topmost surface. For the compositionally perturbed GaAs_1−*x*_Bi_*x*_ system with *x* ~ 0.04, the depth of *d*_opt_ will be further reduced through an increase in *α*^67^,^68^.

On the other hand, the optical penetration depth for the PL experiments will be much larger. PL signals have been detected from deep below the surface, even through a relatively thick GaAs_1−*x*_Bi_x_ epilayers (>300 nm); detecting the efficiently laminating GaAs substrate from GaAs_1−*x*_Bi_*x*_/GaAs structures is a common feature for PL studies of these films^41^,^69^. There are two main reasons for this: (i) photons emitted from the material as luminescence will possess longer wavelengths (lower energy) than the excitation wavelength, and will be able to escape from a larger depth (*α*_out_<<*α*_in_), and (ii) the excited charge carriers may diffuse deeper into the material and recombine (emitting PL) a further distance away from where they were excited. However, the majority of the luminescence emitted will arise from just a few tens of nm from the semiconductor surface and small topographical changes are expected to be sensitively resolved.

Note that under high magnification (x100 objective) employed in our micro-optical experiments the confocal optics of our instrument additionally lowers the magnitude of *d*_opt_ by limiting the detection of emitted photons to inside the focused beam path. While an exact value of *d*_opt_ for the micro-Raman and micro-PL measurements is not defined here, we note that *d*_opt_ will have a finite value when probing the complicated structure of our embedded nanostructure, as well as recognise the impact this has on the types of conclusions that may be drawn from these micro-optical measurements. Polarised Raman analysis is employed to observe experimental trends (Raman selection rules). It is believed that the magnitude of *d*_opt_ here will not significantly stifle the ability to draw conclusions about local phase and relative orientation, from polarised micro-Raman analysis. In contrast, we point out that without moving to shorter excitation wavelengths, this particular experimental constraint indeed renders the micro-PL characterisation in this section strictly *qualitative*.

#### Micro-Raman survey of sample surface

**Figure 6:**
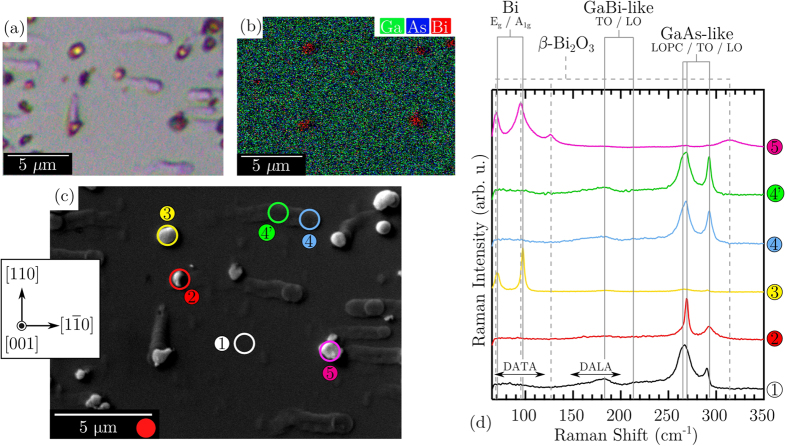
Micro-Raman characterisation of various surface features atop the droplet covered GaAsBi surface. Corresponding (**a**) differential optical (false color) micrograph (**b**) EDS micrograph and (**c**) secondary electron SEM image of the droplet covered GaAsBi surface. In (**c**), local areas of interest are identified and the inset shows the relative crystal orientation, as determined by combining polarised Raman scattering measurements (Fig. 5(a)) and surface energy theory^61^. The red circle next to the scale bar in (**c**) indicates the relative spot size of the optical microprobe for both micro-Raman and micro-PL measurements. (**d**) Room-temperature micro-Raman spectra recorded from locations of the interest depicted in (**c**). Spectra have been normalised and offset for clarity, with the vertical lines corresponding to the frequencies of Bi, *β*-Bi_2_O_3_, GaBi-like and GaAs-like optic modes.

Figure 6(a–c) presents corresponding micrographs exhibiting the full variety of features found on our GaAsBi surface, using optical and scanning electron techniques. The optical image in Fig. 6(a) was recorded using topographically-sensitive differential interference contrast (DIC) microscopy, and again reveals a flat epitaxial surface interrupted by the metallic surface droplets, and the formation of trailing nanotracks. Of the surface features, six typical locations are identified in the SEM image in Fig. 6(c) for detailed treatment here. The segregation of elemental bismuth on the epitaxial surface is readily identified in the EDS micrograph shown in Fig. 6(b), however determining the surface quality and form (i.e. microstructure/orientation, metallic purity, crystallinity, or chemical oxidation) requires a phase-specific technique. Figure 6(d) presents depolarised micro-Raman spectra recorded from the locations indicated in Fig. 6(c), along with the vibrational signatures associated with Bi, *β*-Bi_2_O_3_, GaBi-like and GaAs-like optical modes. By way of alignment with these signatures, we are able to characterise these surface features using a Raman microprobe.

The spectral features and two-mode behaviour exhibited from 1 (smooth droplet-free region) is characteristic of GaAs_1−*x*_Bi_*x*_ for *x* ~ 0.04^50^,^70^. Just below the GaAs-like TO(Γ) frequency, a large damped LO-phonon-plasmon-coupled (LOPC) mode appears, the origins for which in our nominally undoped sample are discussed in detail in ref. 50. According to the Raman selection rules for (001)-oriented zincblende crystal^71^, the LO mode is allowed for certain backscattering geometries, while TO is always forbidden. The weak appearance of the GaAs-like TO mode here indicates the presence of Bi-induced structural disorder and matrix deformation, and a subsequent relaxation of the selection rules. Further, there also exists in the spectrum broadband lifting of disorder-activated transverse (DATA) and longitudinal acoustic (DALA) signatures^72^. The spectrum recorded from the structure at 2 has a very strong TO mode contribution, which is surprisingly large compared to the LO mode, for backscattering from a nominally (001)-oriented growth surface^71^. Such a shift in spectral weighting is indicative of an out-of-plane growth which is significantly misorientated away from the original [001] *c*-axis of the substrate. The strength of the GaBi-like vibrations in the 2 spectrum is also considerably less than that observed in 1, suggesting a substantially less Bi incorporation into this surface location. Further, the LOPC mode is significantly reduced. The unusual character of 2, as well as its crystallographic orientation, is examined in detail later using polarised Raman scattering. The micro-Raman spectrum recorded from the droplet at 3 exhibits two prominent modes at 73 cm^−1^ and 98 cm^−1^, which is consistent with the two first-order optical bands of rhombohedral Bi (A7 structure) corresponding to the *E*_g_ and *A*_1g_ phonon modes, respectively^73^. The frequency, linewidths, and second-order harmonics measured here indicate a crystalline Bi structure. The spectra obtained from the embedded nanowire termination (forming a microdisc^16^) and nanotrack at 4 and 4′, respectively, are very similar, exhibiting a two-mode behaviour comparable to that recorded from 1. These spectra are representative of a number of similarly heavily-overgrown features investigated on the epitaxial surface and confirm the detection of a non-trivial GaAsBi EO layer. Essentially there are only minor differences between spectra 4 and 4′, and 1, which exist mainly in the GaAs-like optical bands; the TO and LO phonons intensify and slightly blueshift, with the strengthening of the TO mode in 4 greater than 4′. While a quantitative interpretation for the symmetry-forbidden TO mode cannot be performed, its relative magnitude is an indicator of local electric field perturbations (structural defects), leading to its appearance. Due to the similar character of 4 and 4′, onward we will focus on location 4 and consider it representative of surface EO nanotrack material. The final topographical feature to be characterised is 5, which exhibits vibrational signatures associated with a room-temperature metastable^74^ form of bismuth oxide (Bi_2_O_3_) in the *β*-phase^75^. Due to the very low melting point of bismuth metal, it has a high tendency to oxidise. Thus, the appearance of bismuth oxide on the droplet-covered surface is not surprising and is not considered any further here.

#### Polarised micro-Raman characterisation

**Figure 7:**
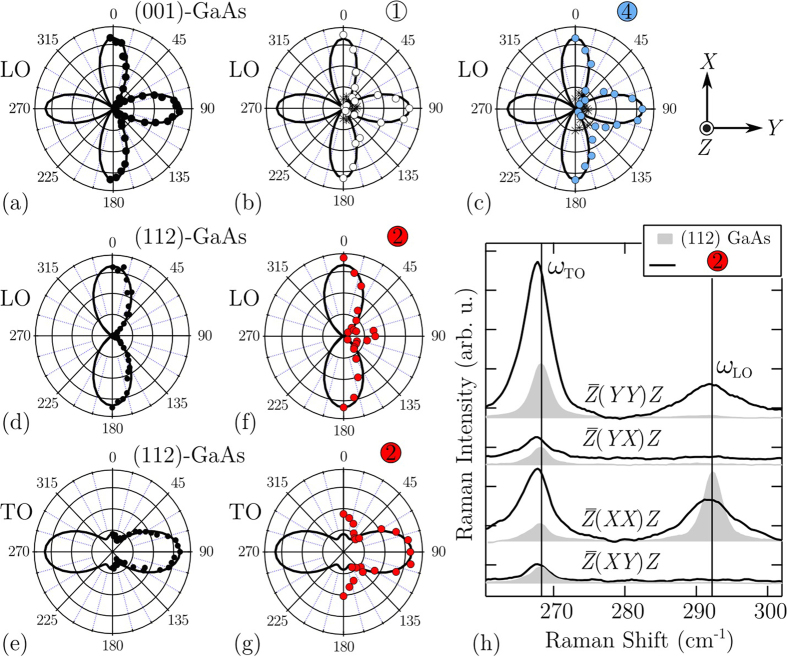
Polarised Raman scattering data recorded from the GaAsBi sample surface. Normalised Raman backscattering polar plots of GaAs and GaAs-like optic modes measured in 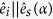 configuration from (**a**) (001)-GaAs single crystal; (**b**) 1; (**c**) 4; (**d**) and (**e**) (112)-GaAs; (**f**,**g**) 2. For the case of plots (**a–c**), the TO mode is forbidden in a backscattering geometry, though the observed intensity relative to the LO mode is represented by the asterisk symbols (close to the origin). The bases of all the polar plots are represented by the coordinate system provided in the top right inset and correspond in the laboratory coordinates. (**h**) Comparison of the selection rules measured from 2 and a (112)-oriented GaAs. The vertical lines in (**h**) indicate the TO and LO frequencies of pure GaAs.

Figure 7 displays polarised first-order Raman scattering data recorded from 1, 2, 4 and, for comparison, (001)- and (112)-oriented GaAs single crystals. The polar data were again measured employing a parallel polarised configuration (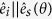) rotated through 0° ≤ *θ* ≤ 180°. For the analysis of (001)-oriented crystal, the (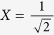
[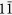
0], 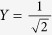
[110], and *Z* = [001]) basis is employed, while for (112)-oriented crystal (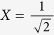
[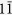
0], 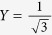
[11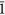], and 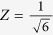
[112]) is adopted. In aid of assessing the polar data in Fig. 7(a–g), the calculated intensities—using the tensors provided by Puech *et al*.^76^—for each mode are also presented (solid lines). To investigate the character of 2, data for the four principal polarised geometries are presented in Fig. 7(h). For comparison, these data are presented along side measurements made on (112) GaAs. As we will see through Raman analysis, a comparison between the two will help in understanding the microstructure of the crystal within 2.

For the case of 1, the results for the GaAs-like polar LO phonon intensity is essentially the same as that recorded from the (001)-GaAs crystal; both experience a periodic LO phonon intensity which cycles every 90° and virtually no TO band. The presence of a weak TO peak that is invariant to changes in *θ* is in agreement with its disorder-activated origins. Comparable polar data is recorded from 4 and indicates the planar nanotrack crystal aligns well with the epitaxial film (and substrate), with no significant misalignment or rotation around the *Z* axis. This is not surprising, given the way in which the VLS-grown nanowire grows from the epitaxial surface and the coherent nature in which the EO crystal is deposited atop the EO/nanowire interface (for example, see image^4^ in Fig. 3(c)). The strength of the disorder-activated TO band in 4 is verified to be stronger and approximately double to that of 1. This simply indicates an elevated level of structural disorder present in the nanotrack, compared to the epitaxial surface, likely originating in the graded boundaries of the structure contained within the optical probe.

As revealed in Fig. 6(d), an initial inspection of the Raman spectrum recorded from 2 suggests the growth of a non-(001) surface. Further, the large weighting of the GaAs-like TO band, relative to the LO band, indicates significant misalignment away from an original [001] growth direction; such as one of the [*hhk*] directions described ref. 76. Examining the polarised Raman data measured from 2 in Fig. 7 allows a simple reduction of these possibilities. First, for light Raman scattered from a (110) crystal, the LO is symmetry forbidden and should be relatively weak, while for the case of a (111) surface, the intensity of TO and LO should be approximately invariant to changes in *θ*, ruling out these planes. However, the polar data does resemble that of a [112]-oriented structure, or perhaps even a higher index plane. As expected for a strained and disordered system, the phonon energies measured here for the GaAs-like optical modes in 2 are redshifted to lower energies, compared to that of a pristine GaAs system. The redshift of the TO phonon frequency (Δ_*ω*Γo_) is considerably larger than the LO frequency (Δ_*ω*Γo_), measured to be Δ_*ω*Γo_ = −0.8 cm^–1^ and Δ_*ω*Lo_ = –0.3 cm^–1^, respectively. This is in contrast to the strain-induced shifts seen from light Raman scattered from a (001) surface, for which a greater shift in the LO band is typically seen^76^. Considering the strain-induced optic mode redshift coefficient values for backscattering from the various [*hhk*] surfaces^76^,^77^, we find that a markedly larger shift in the TO phonon is only seen for plane indices lower than (113). This suggests 2 might be oriented somewhere between [113] and [111].

To further understand the orientation of 2, next we examine its notable deviations from the typical selection rules of (112) GaAs, displayed in Fig. 7(h). Again, a likely explanation lies in the strain state of 2. In Fig. 7(h) the selection rules of the GaAs-like optic modes are comparable (active or non-active) for the 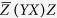, 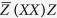 and 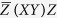 polarised geometries; LO is symmetry-forbidden in the crossed polarisations, confirming the relative orientation of the polarisers versus the crystallographic axis. However, for the 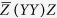 geometry, an activation of the LO phonon is observed. Calculations made by Mailhiot and Smith^78^ for the three components of the strain-induced electric field in our adopted (112) basis show that the polarisation along the *Z* direction is strong, while the in-plane polarisation only contains a large *Y* component^76^. This strain property, along with some likely deviation away from a true [112] direction, may account for this experimental observation.

As established previously, the anomalous character of the optical mode recorded from 2 cannot be attributed to the presence of a local WZ crystal structure, given the defining WZ mode at 255 cm^−1^ 56,57 is not observed. Notably, there is a general retention in 2 for a common 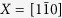, relative to the epitaxial growth. This suggests a non-trivial crystallographic reorientation – due to multifaceting, or otherwise – in the *Y-Z* plane. While a more exact evaluation of the crystal orientation of 2 (with respect to the (001)-oriented substrate/epilayer) or its origins are at present lacking, we simply emphasise the interesting nature of this non-(001)-oriented growth, catalysed in the presence of Bi surface droplets. Within the context of growing device-quality GaAsBi materials, such a finding is highly undesirable. Next we examine the important light-emitting properties of these surface features.

#### Micro-photoluminescence characterisation

EDS and micro-Raman characterisation of the nanotracks have already revealed the presence of localised inhomogeneities in the structure and composition of the Bi covered epitaxial surface. In particular, the surface nanotracks are established by the epitaxial the burial of subsurface GaAs nanowires and possess a reduced structural quality and a lower Bi concentration. Thus, one would expect then the surface features to exhibit optical changes and a smaller Bi-induced bandstructure modification through PL, i.e. the desirable alloying-induced bandgap reduction is smaller. Furthermore, the relationship between the micro-PL peak energy (*E*_PL_) and the Bi concentration reported by Lu *et al*.^41^ provides a means of quantifying this change in composition, through PL analysis. In terms of the scientific and technological drivers for GaAs_1−*x*_Bi_*x*_ alloys, their bandgap energy and light emitting properties are by far some of the most important. As well as providing the spatial resolution required to study the features atop our GaAsBi sample, the focusing optics in micro-PL can achieve extremely high excitation power densities (*I*_pump_), offering more insightful and dynamical forms of the PL measurement—pump-dependence studies.

**Figure 8:**
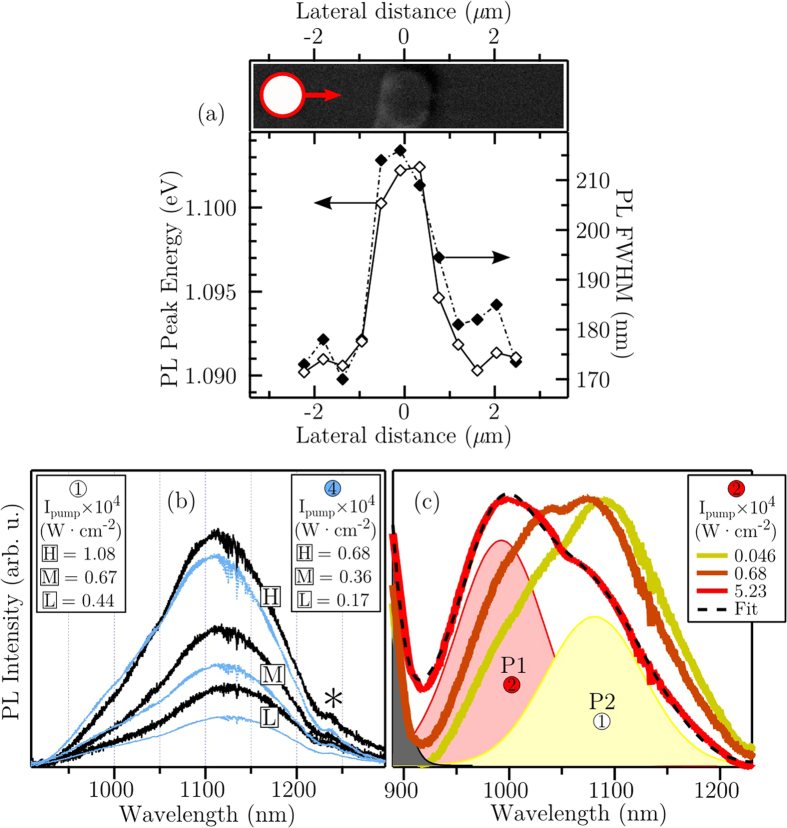
Micro-PL characterisation of GaAsBi sample surface. (**a**) PL peak energy and FWHM of micro-PL spectra recorded using a relatively low power density (*I*_pump_ = 5 × 10^2^ W.cm^−2^) along a line scan across 4. The top micrograph is an SEM image of 4 showing the path taken by the scan and the relative size of the microprobe. (**b**) Micro-PL spectra recorded from 1 and 4 for three differing laser power densities (*I*_pump_ = low [L], medium [M] and high [H]), exhibiting typical spectral changes; signal intensification and a blueshift in peak energy. Here ‘*’ indicates a spectral artefact. Due to the difference in the absolute PL signal recorded from the two locations, the three laser powers ([L], [M] and [H]) used to measure the PL spectra from 1 and 4 are not equal (see plot keys for comparison). (**c**) Three normalised PL spectra recorded from 2 covering two orders of magnitude of *I*_pump_, showing two resolved Gaussian peaks, P1 and P2, originating from feature 2 and from the surrounding epilayer, labelled 1. All data were recorded at room temperature.

Figure 8(a) presents the measured room-temperature PL peak energy and FWHM of low- *I*_pump_ spectra obtained from a line scan across 4. The distinct shift here in E_PL_ and the FWHM measured from 4 clearly demonstrates a local discontinuity in the properties of the surface epitaxy in the presence of VLS-driven Bi droplets. The blueshift in energy may be understood as a local decrease in Bi concentration within the EO which forms the nanotrack, while the rise in FWHM has two possible origins: (i) a density of states tail manifested by relatively high structural/mechanical disorder within the structure of the probed volume, and (ii) the superposition of an unresolved emissions from the compositional boundaries within the probe volume.

Figure 8(b) displays micro-PL spectra recorded from locations 1 and 4 for three increasing values of *I*_pump_. A steady increase in the overall PL signal is seen for a rising *I*_pump_, due to the brighter pump light producing more electron-hole pairs for radiative recombination. Interestingly, we also see the PL peak energy shift to shorter wavelengths for increasing irradiance. Lu *et al*.^41^, as well as other investigators^79^,^80^, have already reported this type of excitation-dependence for PL signals measured from GaAs_1–*x*_Bi_*x*_. Such dependencies are signatures often seen in high-mismatched alloys involving localised states and parallels may be drawn here to the PL dynamics of the GaAsN system^81^,^82^. The mismatch between Bi (and N) with As induces an unusual optical response by introducing an impurity-like localised character in the host GaAs band structure^83^, as a consequence of its hybridisation with the Bi-induced (N-induced) wave function.

Figure 8(c) focuses on the more complex PL spectra recorded from 2. In the normalised PL data two separate Gaussian peaks of differing energies are resolved and, for higher excitation intensities, the spectra display a similar blueshift in the peak energies. An analysis of the pump-dependence indicates a dominant high-energy emission arising from 2 (P1), with a second PL peak (P2) exhibiting a character (peak energy and FWHM values) similar to that measured from 1, and is attributed to PL emitted by the underlying/surrounding epitaxial layer.

**Figure 9:**
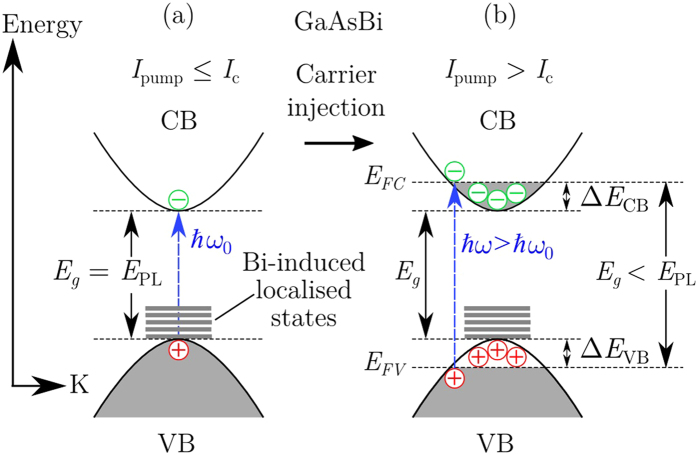
Schematic illustration of band filling effect in electronic band diagram of GaAsBi. (**a**) The measured *E*_PL_ equal to the intrinsic direct bandgap of GaAsBi for *I*_pump_ below a critical excitation intensity, *I*_c_. (**b**) For *I*_pump_ above *I*_c_, the “apparent” optical bandgap is widened due to a large carrier injection and recombination occurs between the Fermi levels in the CB (*E*_FC_) and VB (*E*_FV_); between states which no longer lie at their respective band edges.

**Figure 10:**
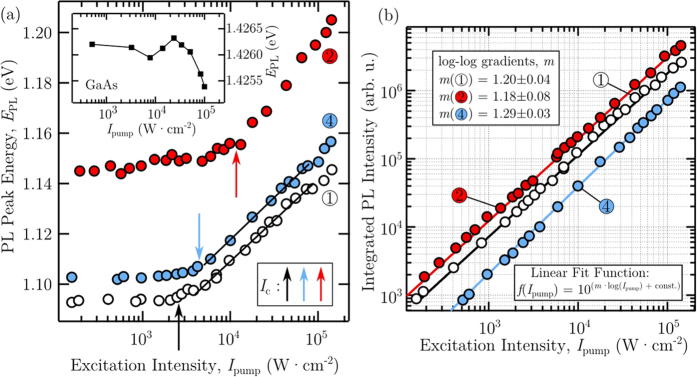
Analysis of excitation intensity-dependent micro-PL data. (**a**) Measured room-temperature micro-PL peak energy dependence on excitation intensity, *I*_pump_. The arrows indicate the differing critical intensities (*I*_c_) measured from locations 1, 2 and 4, where a strong monotonic blueshift is seen in the PL peak energies due to band filling. The inset shows similar measurements made on pure GaAs. (**b**) Room-temperature PL intensity, *I*_PL_, recorded from 1, 2 and 4, as a function of the pump intensity, *I*_pump_.

In Fig. 8(b,c), the shift in the PL peak energy under high illumination is due to the band filling effect; this effect leads to a blueshift in the observed luminescence for increasing photo-induced carriers. Figure 9 shows a schematic illustration of this phenomena in relation to the GaAsBi system and considers both possible photo-induced perturbations of the quasi-Fermi energy levels for the CB (Δ*E*_CB_) and VB (Δ*E*_VB_). For the case of GaAsBi alloys, this effects is known to be exaggerated^41^ by the high level of Bi-induced localised states near the VB maximum^27^,^83^, causing a degenerate charge distribution in this region. Consequently, due to the Pauli exclusion principle, radiative recombination between occupied states in CB and VB are forbidden and the possible transitions are pushed to higher energies. Considering the VB edge in GaAs experiences most of the change from Bi incorporation^83^,^84^,^85^, it is reasonable to assume that Δ*E*_VB_ is the dominant contributor to the pump-dependent shift in *E*_PL_. For comparison, in an attempt to optically induce a Burstein-Moss shift (band filling with only states close to the CB minimum being populated) in pure GaAs, high *I*_pump_ measurements show very little shift in *E*_PL_ over the pump intensity range studied here. This data is shown in the inset of Fig. 10. In fact, one sees a minor redshift, rather than a blueshift, for an extremely high *I*_pump_. This is likely due the local temperature increase within the high-intensity microprobe, which would slightly reduce the bandgap energy.

To study in detail the interesting excitation dependence of probed locations 1, 2 and 4, micro-PL spectra were measured with *I*_pump_ values over approximately three orders of magnitude: 1.5 × 10^2^ to 1.4 × 10^5^ W.cm^−2^. Figure 10(a) presents the excitation intensity dependence of the PL peak energy measured from these locations. For low *I*_pump_, the shape and energy of the emission from all three locations remains stable over an order of magnitude. The peak energy measured under low irradiance accurately portrays the intrinsic bandgap energy of the probed material. Consistent with the extensive compositional analysis of our epilayers and the data contained in Fig. 8(a), we see a slight blueshift in *E*_PL_ recorded using a low *I*_pump_ from the EO covered 4, compared to 1, as well as a much larger blueshift in 2. Both of the shifts in PL peak energies measured from 2 and 4 are presumed to originate from a relative reduction in the local Bi molar fraction contained in these locations.

On the other hand, above a critical excitation intensity threshold in Fig. 10(a) (indicated by *I*_c_), we observe a monotonic increase in the PL peak energy. With rising *I*_pump_, more photo-excited charge carriers are created and the energy for recombination is broadened, increasing *E*_PL_. Over the excitation range studied in Fig. 10(a) a relatively large total shift of ~−50 meV is experienced.

In quantifying the rate of blueshift observed in the PL peak energy, Lu *et al*.^41^ showed that the thermalisation depth *ε* experiences the following pump-dependence:


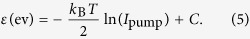


Here *T* is temperature, *k*_B_ is the Boltzmann constant and *C* is a constant. Employing Eq. 5 we apply a linear fit to traces 1 and 4 in Fig. 10(a), yielding comparable gradients. The slope of the fitted lines here is proportional to −*k*_*B*_*T/2*, permitting the calculation of temperature, *T*, from this relation. The temperatures derived in this way from traces 1 and 4 are 328 K and 335 K, respectively, and are consistent with the recording of these data at room temperature.

Interestingly, the values of *I*_c_ in Fig. 10(a) differ between the different micro-probed locations. Factors influencing the exact value of *I*_c_ are vague, though Lu *et al*.^41^ suggested an inverse relationship with the bismuth content, and thus the density of Bi-related localised states. Here we see a similar compositional trend (Bi content inferred from PL peak energy measured at low *I*_pump_) across three different locations on one GaAsBi surface; the value of *I*_c_ is lowered for materials exhibiting PL peak energies with larger Bi-induced reductions.

Investigating the dependence of the integrated PL signal (*I*_PL_) on the excitation intensity can yield insights into the dominant recombination mechanism present within the optical microprobe^86^,^87^. The presence of dominant nonradiative recombination is closely linked to the density of defects that provide mediating recombination avenues. Typically the measured PL intensity is proportional to the injected carrier density through a power law^86^. For a fixed microprobe volume, therefore, the integrated PL intensity *I*_PL_ will scale with the excitation intensity by^86^,^87^


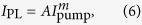


where *A* is a scaling factor and, through theoretical considerations, exponent *m* will acquire a value typically between 1 and 2, depending on the proportions of radiative and nonradiative recombination. For *m* = 1, excitonic radiative recombination dominates. However for m = 2, Shockley-Read-Hall recombination occurs^86^ relating to the presence of nonradiative deep-level traps, induced through defect states, which provide a shunt path for the carriers.

Figure 10(b) displays a log-log plot of the excitation dependence of the *I*_PL_ measured from 1, 2 and 4. The straight line fits are made using the equation in the inset and yield the values of *m* displayed. Three different exponent values *m* are acquired from the locations studied on our GaAsBi surface. For the case of the epitaxial film, a value of *m*(1) = 1.20 ± 0.04 is measured and suggests radiative recombination to dominate. Agreeing with the micro-Raman characterisation of the nanotrack/nanodisc, the relatively higher value of *m*(4) = 1.29 ± 0.03 indicates an increase in the defect density within the probed region, leading to a greater proportion of carrier recombining through Shockley-Read-Hall processes. Conversely, the exponent value *m*(2) = 1.18 ± 0.07 is comparable to *m*(1), if only marginally less. This suggests less defects may be serving as nonradiative recombination centres within 2. From the PL peak energy measured from 2, a significant reduction in the Bi content is expected to exist and may account for a relative increase in the structural quality of the crystal. While it is difficult to accurately isolate the causal influences that locally vary *m*, these findings further exhibit non-trivial inhomogeneities across the Bi-droplet covered surface. In this case, an inhomogeneity in a very important optical parameter related to the light emitting efficiency of the semiconductor.

## Conclusion

In conclusion, we have investigated the surface effects of VLS-driven Bi droplets formed during the growth of a MBE-grown GaAsBi epilayer. Cross-sectional electron microscopy of our epitaxial structure reveals the presence of a grading step and that self-aligned “nanotracks” trailing the Bi surface droplets possess a complicated subsurface morphology. The details of the compositional profile of the film cross-section were detailed through a combination of HRXRD, PL and EDS analyses. Specifically, the epitaxial overgrowth of embedded planar nanowires is shown to give rise to the surface trails of reduced Bi alloying (~25% less), relative to the surrounding epitaxial layer. The phase properties of our film were investigated and the VLS mechanism underpinning the formation of the nanowires and nanotracks trailing the Bi surface droplets was presented, as well as on the nature of nanotrack self-alignment. Through analysis of polarised micro-Raman scattering measurements, the nanotrack EO crystal is shown to align with the substrate and possess elevated structural disorder, relative to the epitaxial film. Large inhomogeneities in the optical properties—bandgap energy and light emitting efficiency—are found to occur over the nanotrack covered surface, suggesting that the surface may play an important role in the development of future devices based on this material. At the very least the surface properties must be considered when designing and fabricating optical devices.

## Additional Information

**How to cite this article**: Steele, J. A. *et al*. Surface effects of vapour-liquid-solid driven Bi surface droplets formed during molecular-beam-epitaxy of GaAsBi. *Sci. Rep.* **6**, 28860; doi: 10.1038/srep28860 (2016).
